# Dynamic colour change as a signalling tool in bluelined goatfish (*Upeneicthtys lineatus*)

**DOI:** 10.1002/ece3.10328

**Published:** 2023-08-25

**Authors:** Louise Tosetto, Nathan S. Hart, Jane E. Williamson

**Affiliations:** ^1^ School of Natural Sciences Macquarie University Sydney New South Wales Australia

**Keywords:** communication, foraging signal, goatfishes, Mullidae, physiological colour change

## Abstract

Many animal species can rapidly change their body colouration and patterning, but often the ecological drivers of such changes are unknown. Here, we explored dynamic colour change in the bluelined goatfish, *Upeneichthys lineatus*, a temperate marine teleost species. *Upeneichthus lineatus* can change in a matter of seconds, from a uniform white colour to display prominent, vertical, dark red stripes. Initial observations suggested that rapid colour change in *U. lineatus* was associated with feeding and may act as a signal to both conspecifics and heterospecifics that are frequently observed to follow feeding goatfish. Field observations of the colour and behaviour of individual *U. lineatus* were collected to (1) document the repertoire of behaviours that *U. lineatus* displays and categorise associated colour patterns; (2) quantify the speed of dynamic colour change; (3) establish the context in which *U. lineatus* changes colour and pattern; and (4) test whether the behaviour of follower fishes is influenced by colour patterning or specific behaviours of the focal goatfish. We found that *U. lineatus* changed colouration from white to the red banded pattern in less than 10 s. The key driver of rapid colour change in *U. lineatus* was feeding, particularly when the fish fed with its head buried in sediment. Conspecific followers were most likely to be white in colour and adopt searching behaviour, regardless of the focal fish colour or behaviour. Other species of follower fish spent significantly more time following *U. lineatus* that were displaying dark red stripes when searching or eating, implying the red stripes may be an interspecific signalling mechanism. Our findings indicate that rapid colour change in teleost fish may be used for social communication and may provide *U. lineatus* with increased protection from predation when feeding via a safety‐in‐numbers approach.

## Graphical abstract

Bluelined goatfish can dynamically change their body pattern and colour from a neutral white to a dark red banded pattern under 10 s. Goatfish are more likely to adopt a dark red banded pattern when actively feeding. Furthermore, associated fishes spend more time with goatfish displaying the red banding. It is likely bluelined goatfish use rapid colour change as a social signalling tool.

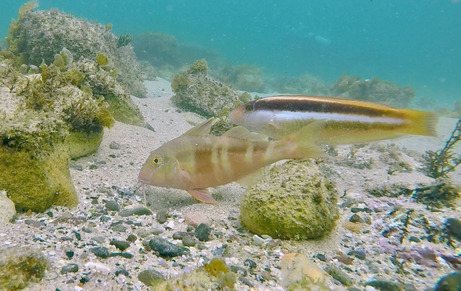

## 1 INTRODUCTION

A spectacular assortment of integumentary colours and patterning are exhibited across the animal kingdom, and some of the most remarkable displays occur in marine organisms. Animal colour is not always fixed and many invertebrates and poikilothermic vertebrates, including fish, change their colouration and/or patterning depending on their developmental or environmental context (Duarte et al., 2017; Figon & Casas, 2018; Umbers et al., 2014). Some animals alter their colour over long periods of time (days to months) in response to seasonal changes or ontogenetic shifts (Cortesi et al., 2016; Leclercq et al., 2010) in a semipermanent process called morphological colour change (Nilsson Sköld et al., 2016). In other animals, the shift in colour and/or pattern can be much quicker, sometimes in seconds. Such rapid or physiological colour change is rarely permanent and provides individuals with phenotypic plasticity in their response to environmental conditions (Nilsson Sköld et al., 2013). In the marine environment, multiple drivers have been linked to rapid changes in colour and patterning across diverse organisms, with camouflage and sexual signalling the most widely reported. Animals use camouflage to conceal themselves to reduce detection and recognition by predators (Duarte et al., 2017; Stevens, 2007). Camouflage by background matching is a particularly effective defence if an animal can blend in with its surroundings, but there are a number of other strategies used to reduce detection, including disruptive colouration, motion dazzle and motion camouflage (Stevens & Merilaita, 2009). Camouflage via rapid colour change is well documented in cephalopods (Brown et al., 2012; Hanlon, 2007; Hanlon et al., 2009) and other marine invertebrates (Green et al., 2019; Hultgren & Mittelstaedt, 2015; Stevens et al., 2013). It has also been widely reported in several flatfish species (Akkaynak et al., 2017; Ramachandran et al., 1996; Ryer & Olla, 1992), Nassau groupers (Watson et al., 2014) and rockpool gobies (*Gobius paganellus*; Stevens et al., 2014).

In sexual signalling, rapid colour change is used as a conspicuous signal where distinct colour patterning can be used to attract and to advertise conspecifics, particularly in the presence of competitors (Price et al., 2008). Carotenoid colouration (yellow, orange and red) is used in the marine environment to advertise quality (Svensson & Wong, 2011). For example, female two‐spot gobies (*Gobiusculus flavescens*) develop bright orange bellies in the breeding season to attract male mates (Svensson et al., 2006), and the aptly named pink‐belly wrasse (*Halichoeres margaritaceus*) advertises its readiness to spawn by shifting its usual abdomen colour to red (LaPlante, 2015, 2017). However, sexual signalling in the marine environment can also involve purely achromatic signals. Female pipefish (*Syngnathus typhle*), for example, display a temporary ornament of evenly spaced vertical black bars to signal their competitive ability to other females as well as attract male pipefish (Berglund & Rosenqvist, 2001). More recently it was discovered that lined seahorses (*Hippocampus erectus*) will alter luminance when interacting with their pair mate (Mederos et al., 2022). Kelp bass (*Parablabrax clathratus*; Erisman & Allen, 2005), sand bass (*Paralabrax maculatofasciatus*; Miller & Allen, 2006) and fishgod blennys (*Malacoctenus ebisui*; Galván‐Villa & Hastings, 2018) are also all reported to adopt highly contrasted body patterning to advertise their reproductive status.

Rapid colour change is also used for other forms of inter‐ and intraspecific communication. In a mutualistic interaction between cleaner shrimp and reef fish, several fish have been observed to rapidly shift their body colour to appear darker to the shrimp, indicating their willingness to be cleaned (Caves, Green, & Johnsen, 2018). Some fish have a repertoire of body patterns that are scenario‐dependent in use. For instance, bullethead parrotfish (*Chlorurus sordidus*) employ three key achromatic patterns to switch between: (1) a ‘non‐specific’ and uniformly dark colouration that is often displayed when feeding; (2) a bullseye on the caudal fin frequently shown by non‐schooling juveniles and thought to reduce mortality through predation given its resemblance to an eyespot; and (3) longitudinal stripes that aid predator evasion by breaking up the outline of the animal, particularly when displayed in a large school of fish (Crook, 1997). The bluestriped fangblenny (*Plagiotremus rhinorhynchos*) is an aggressive mimic that can rapidly change colour to imitate several different reef fish, depending on the availability of other species nearby (Cheney et al., 2009). While some mechanisms and behaviour repertoires associated with visual communication are well documented, behavioural drivers of colour change for other species are not yet understood. Reflective stripes on the nose of paradise whiptail (*Pentapodus paradiseus*) flash through four different colours in less than 3 s (Mäthger et al., 2003), and the flashing tilefish (*Hoplolatilus chlupatyi*) can rapidly change its body colour from blue to red (Goda, 2017). In both examples, it has been demonstrated that the fish rapidly change colour on adrenergic stimulation; however, the behavioural function of this change has not been investigated. Similarly, the hogfish (*Lachnolaimus maximus*) exhibits dynamic changes in both integument colour and patterning (Schweikert et al., 2018), but the drivers of this exhibition have not been empirically tested.

**FIGURE 1:**
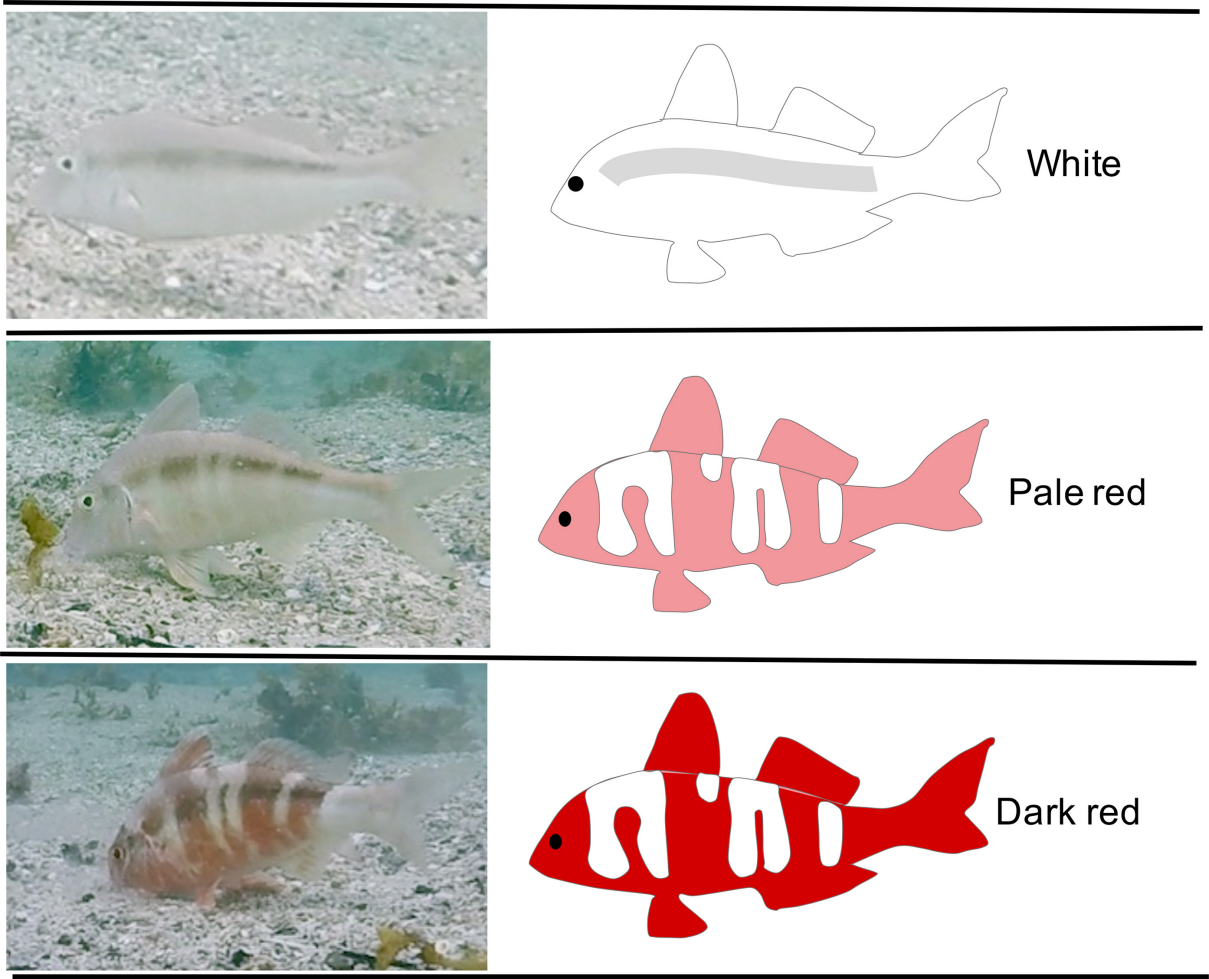
Body colour patterns of the bluelined goatfish (*Upeneichthys lineatus*). The three different colour patterns exhibited by bluelined goatfish. These images are from the same goatfish taken over a 30‐s interval.

Bluelined goatfish (*Upeneichthys lineatus*), which occur in coastal waters along the southeastern coast of Australia, have also been observed to rapidly change both colour and pattern (Tosetto; personal observation). Typically, *U. lineatus* change from a pale buff/white colour to a prominent, vertically striped, red pattern in seconds. There are anecdotal reports these fish adopt a red colour when resting at night, but during the day they regularly transform their body colour and pattern when foraging, seemingly making them more conspicuous (Figure 1). *Upeneichthys lineatus* are zoobenthivores that feed in a similar fashion to other goatfish. Using a shovelling action, they bury their snout in the substrate to search for prey, with their body position tilted by more than 30 degrees to the horizontal (Krajewski et al., 2006). It is likely that this feeding position restricts their visual field and compromises their capacity to escape. Similarly, rabbitfish (f. Siganidae) often feed with their heads down in substrate and crevices, making predator detection difficult. Rabbitfish that work in pairs and alternate between feeding and vigilance appear to benefit by increasing foraging efficiency (Brandl & Bellwood, 2015). Rather than work in pairs, it is possible that colour change in *U. lineatus* attracts other fish, which may result in increased vigilance and improved foraging. Conspecifics and a host of other fish species (‘follower fish’) are regularly seen to follow feeding goatfish. Furthermore, *Upeneichthys lineatus* can likely discriminate colours at longer wavelengths where the red elements of their body colouration have high reflectance, and it is possible that this provides advantages in distinguishing visual signals from conspecifics (Tosetto et al., 2021). Studies suggest that the key driver of these associations is the feeding behaviour of goatfish, which increases prey availability for follower fish (Sazima et al., 2006). But to date, studies assessing follower associations with goatfish have been undertaken on tropical coral reefs and not considered whether colour change promotes these associations. Given temperate system processes often differ from tropical systems, and that *U. lineatus* can rapidly change colour, it is also possible that follower fish are responding to the colour and pattern shifts exhibited by *U. lineatus* rather than solely their foraging behaviour.

In this study, we assessed the drivers of daytime dynamic colour change in *U. lineatus* and the responses of follower fish to that colour change. To establish the range and speed of dynamic colour change, we documented the different colour morphs and patterns adopted by *U. lineatus* and the rate that they shifted between these states. Next, we investigated the context in which *U. lineatus* changed their colouration and patterning. Using in situ recordings, to establish whether rapid colour change in *U. lineatus* is a background matching strategy, a response to a particular behaviour, or a combination of both, we assessed whether the colour adopted by *U. lineatus* was related to the underlying substrate and/or their behaviour. We explored follower fish associations in relation to focal goatfish by determining whether the following times of both conspecifics and heterospecifics were influenced by a specific behaviour or colour of the focal goatfish, and we explored whether following fish (both conspecifics and heterospecifics) changed colour or behaviour in response to the focal fish. We hypothesise that *U. lineatus* will rapidly change colour when undertaking a particular behaviour, specifically feeding, rather than in response to the underlying substrate. Given that *U. lineatus* appear to be using the red body pattern when feeding, we predict that both conspecifics and heterospecifics will spend more time following the focal goatfish when it is displaying the red colouration. Further, if the red body pattern is indeed a foraging signal, we expect to see an increase in the searching and feeding behaviour by follower fish when the focal goatfish is red.

## 2 METHODS

### 2.1 Field recordings

This study was minimally invasive and complied with the Australian Code for the Care and Use of Animals for Scientific Purposes. The study design was assessed and approved by the Macquarie University Ethics Committee (ARA 2016/020).

To establish drivers of colour change in situ, we followed and filmed *U. lineatus* in 3‐min sessions. Data were collected during the morning (09:00–12:00) between November 2016 and May 2017 using SCUBA. Recordings were made at depths of 5–10 m at three temperate locations in Sydney, Australia, where *U. lineatus* occur regularly: Gordons Bay (33°54′56.66″ S, 151°15′47.26″ E), Shelly Beach (33°48′0.36″ S, 151°17′47.31″ E) and Bare Island (33°59′29.17″ S, 151°13′59.25″ E). These areas are adjacent to rocky shorelines with predominantly sandy bottom interspersed with rocky reefs. As it has been previously reported that *U. lineatus* feeds predominately on sandy substrate, while remaining close to the shelter provided by rocky reefs (Ross et al., 2007), we assumed that individuals in these habitats were likely to feed.

Videos were taken by experienced divers, who recorded between two and eight sessions during each dive. Divers used GoPro™ Hero (2018) video cameras attached to a 1 m‐long PVC pole. All videos were shot in 1080p resolution, at 30 fps and with protune turned off. The GoPro was kept 0.5–1 m away from the focal fish, meaning that the diver was always 1.5–2 m away from the focal fish. During each dive, the first adult goatfish (>20 cm) spotted by a diver was followed. The fish was approached slowly by the diver to 1.5 m and a recording was made if the fish did not obviously alter its behaviour or location in response to the diver. In most cases, goatfish did not appear to change behaviour on a diver's approach. Moreover, an initial pilot study showed no differences in goatfish activity, which included feeding behaviour, between recordings where divers remained at either 1 or 5 m distance from the focal fish (data not shown). Given this, it was decided that a diver remaining 1.5 m from the goatfish was a suitable distance from which to record its behaviour. During recording, the diver would track the fish at a constant distance for three continuous minutes, ensuring that the focal fish remained in the centre of the video. At the end of filming the diver would move to different area of the reef so that a new individual could be located. To avoid filming the same fish twice we used only two divers on each dive that recorded in diverse areas of the site. Further, we recorded at multiple locations to reduce the likelihood of recording the same fish twice. Where possible, individuals were followed for three continuous minutes but in the case that the fish was lost or it hid for more than 30 s the recording was stopped and a new fish was identified. All effort was made to ensure that the divers did not disturb the fish or surrounds.

### 2.2 Establishing behaviours

A total of 35 individual *U. lineatus* were followed. Videos were first assessed to obtain a summary of *U. lineatus* behaviours. Footage was examined in its entirety with the commonly observed behaviours detailed and described in Table 1. The more frequently observed *U. lineatus* behaviours were then binned into three broad behavioural categories: travelling, searching and eating. Film clips showing representative examples of these behaviours are displayed in Videos [Link], [Link], [Link]. In addition to this repertoire of behaviours, three body colour patterns were documented. The shift between these body colour patterns was rapid but continuous (see Video S4 for an example of *U. lineatus* colour change). The white (neutral) phase was defined as a pale buff/white colour with a horizontal darker lateral band. When goatfish were in the white phase, no vertical banding was evident. The pale red phase was defined by the appearance of a vertical striped patterning, often a light red or pink colouration with the horizontal dark lateral band still distinguishable on the body of the fish. The dark red phase was described as a distinctive dark red vertical banding where little lateral banding was present (Figure 1).

**TABLE 1:** Behaviours observed for bluelined goatfish (*Upeneichthys lineatus*) from in situ recordings.

Behavioural category	Detailed behaviours	Description
Travelling	Directed swimming	Rapid movement in a direct manner to another location. Fish is located in mid‐water column well above the substrate. Rapid tail beats
	Wandering	Slow or moderate swimming. Fish is located just above substrate but not in contact with it. Low frequency tail beats
	Chasing	Accelerated swimming while pursuing or being pursued
	Nose‐down swim	Directed movement, nose down and swimming towards the substrate
	Chafe	Quick turn on side against substrate
Searching	Exploring	Moving across the substrate but not in a direct manner and often in an ‘S’ pattern. Substrate stops and checks occur every 10–30 cm using barbels
	Focused search	Intensive prey search. Fish remains in one location and regularly turns 180–360°, placing barbels into the sediment every 1–2 s
Eating	Feeding	Nose down with barbels deep in sediment, often the tip of the snout and eyes are also buried in sediment. Plumes of sediment produced
	Chewing	Stationary individual moving its head in and out of the sediment. Often appearing to chew prey items. Often follows feeding behaviour
	Rapid turn	Rapid turn of head by an individual during feeding, potentially catching escaping prey that may have been disturbed
	Rapid vertical swim	Rapid vertical swimming while chewing. A behaviour often interspersed with feeding/chewing and may be to catch escaping prey items

### 2.3 Quantifying body colour patterns

**FIGURE 2:**
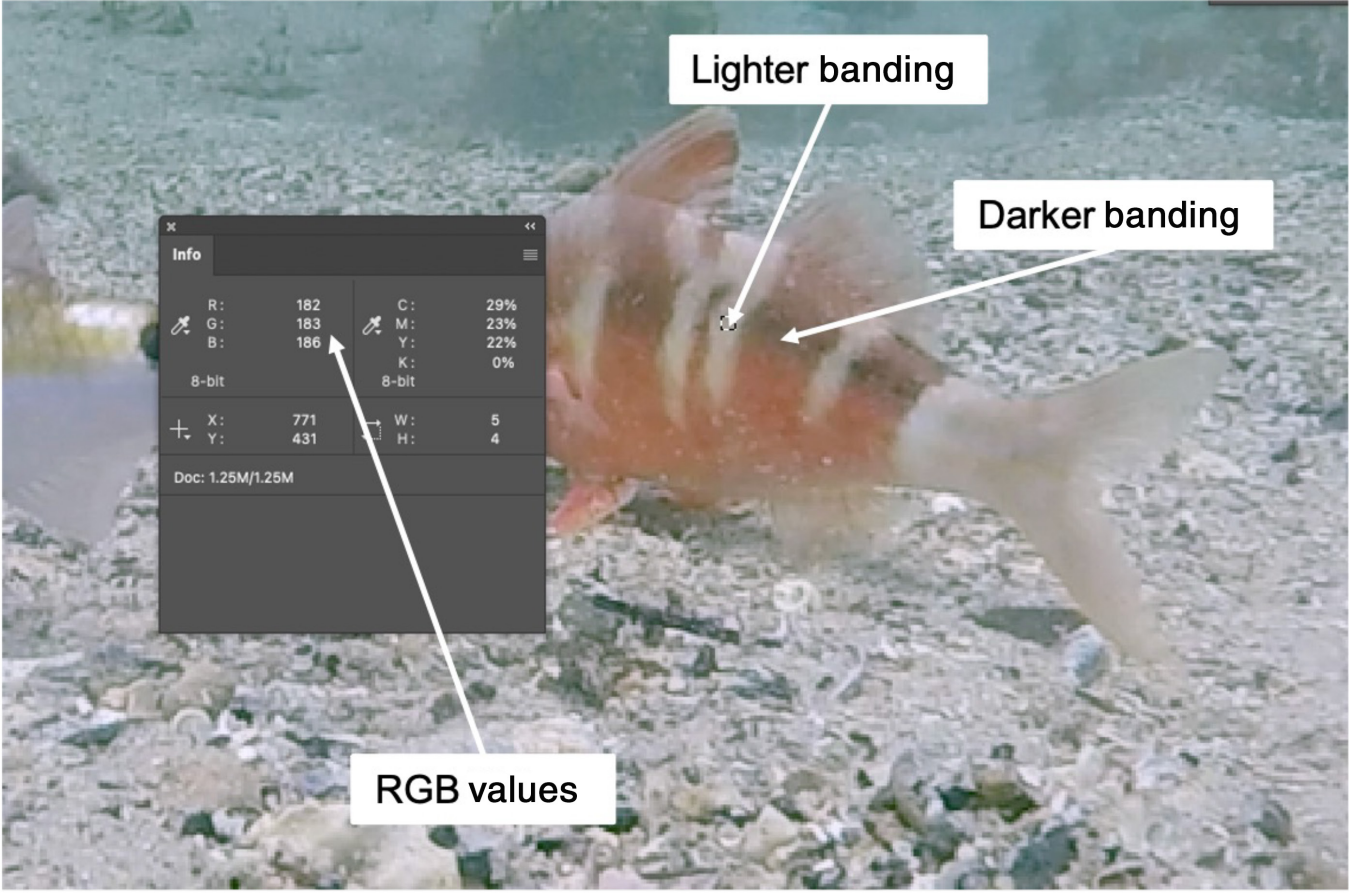
Calculating contrast values for goatfish banding in Adobe Photoshop. Example of obtaining RGB values for relative luminance calculations for the light and dark banding via the info panel in Adobe Photoshop.

To quantify the three different goatfish colour patterns, we measured their internal contrast using colour contrast ratio ranges. Colour contrast ratios, the luminance ratio of the brightest shade to the darkest shade, are properties of digital display systems and calculated using relative luminance. In digital imaging coding systems, such as sRGB, which is used in desktop graphics, reference black and white values correspond to integer values such as 0 and 255 (Poynton & Funt, 2014). Assuming sRGB encoding, the Web Content Accessibility Guidelines (WCAG) 2.1 define the relative luminance of a colour as *L* = R*0.2126 + G*0.7152 + B*0.0722 where *L* is relative luminance, and *R*, *G* and *B* relate to the intensity values for each of the RGB (red, green, blue) components in a colour image. To determine the contrast ratio range for each colour pattern, we first obtained two relative luminance values from 630 frames of video footage (every 10 s). Frames were imported into Adobe Photoshop 2021 (v 22.4.2) and the colour working space was assigned to sRGB via the colour settings in Adobe Photoshop. Using the ‘Info’ panel, we calculated relative luminance using the RGB values for the lighter (L1) and darker band (L2) for each fish (Figure 2). When displaying a white (neutral) colouration, we measured luminance in two places where the bands would usually be apparent (Appendix S1). Wherever possible, we obtained measurements from the same red and white bands towards the middle of the fish. In the event that a fish was orientated differently (12 frames), we took measurements of similarly light and dark banding towards the tail of the fish. To calculate contrast ratios, we used the equation (L1 + 0.05)/(L2 + 0.05) whereby L1 and L2 are the relative luminance for the lighter and darker bands, respectively (Sik‐Lányi, 2012). Contrast ratio values were obtained for the fish banding across a range of environments and conditions with ratios ranging from 0.98 to 2.15 for all fish. We determined arbitrarily that the contrast values for each colour display, were white 0.98–1.04, pale red 1.05–1.19 and dark red > 1.20. These values were used in subsequent video analysis to remove any observer bias in relation to the goatfish body colour pattern.

#### 2.3.1 Rate of colour change in *U. lineatus*

To obtain the rate at which *U. lineatus* changes body colouration, videos were watched at half speed in VLC media player (Version 3.0.16). From the 35 videos recorded, there were 38 occurrences of rapid colour change. Goatfish appeared to rapidly shift colour from white to pale red when they stopped swimming and searched the substratum. The rapid change from pale red to the dark red distinct banded pattern mostly took place when the fish appeared to locate a food patch and actively ate. At the commencement of each colour change event, times were recorded at the point that the fish was white, when it displayed a pale red colouration and again when the fish had adopted the distinct dark red banded patterning with no lateral band visible. Only colour change events where individual *U. lineatus* changed entirely from white to dark red were recorded, such colour change events could take place more than once during one 3‐min track. To determine whether the rate of colour change was similar for individuals from dark red back to white, the time for the fish to shift back to the neutral white colouration following a colour change event was also recorded. Here, the final moment that each fish displayed the distinct dark red banded pattern was recorded and then the time was recorded again the moment it turned pale red and/or white.

### 2.4 Video annotation

Video footage was analysed by an independent observer trained in the behavioural categories and colour morphs of *U. lineatus* but naïve to the overall research questions. Each 3‐min video was watched at half speed using VLC media player and information recorded as per the study objectives. At each 5‐s interval, the behaviour (travelling, searching or eating) and colour (white, pale red or dark red) was recorded for the focal goatfish. The substrate underlying the goatfish (sand, patchy algae or rocky reef) was also recorded at each 5‐s interval. A second observer confirmed the colour morphs that were recorded using the contrast values outlined above. We also recorded all individuals of other fish species (conspecifics or other associated fish species) that actively interacted with or followed the focal goatfish within a radius of approximately 1 m. At each 5‐s time interval each individual follower fish, as well as its behaviour, was recorded. If the follower fish was a conspecific, we recorded its behaviour (travelling, searching and eating) and colour (red, white and pale) in the same manner as the focal goatfish. The behaviours of other follower fishes were categorised in a slightly different manner to that of the focal fish. We recorded travelling behaviour (travelling past with no apparent interaction with the focal fish), eating behaviour (physical feeding from sediment adjacent to the focal fish) and whether they were attracted to the focal fish. Rather than searching for food in the same manner as goatfish, follower fish would regularly demonstrate interest in the focal fish by changing swim direction towards the focal fish, or by closely following the focal goatfish, and it was these interactions that we logged as attracted.

### 2.5 Statistical analyses

#### 2.5.1 Context of *U. lineatus* colour change

All statistical analyses were completed in R (R Core Team, 2022). We tested whether the behaviour or the substrate underneath the goatfish influenced its body colouration by fitting a cumulative link mixed model (clmm) using goatfish colour as an ordinal response term. In the model, the dark red colour was considered the highest ranked response, followed by pale red and then white as progressively lowest ranked response. Focal fish behaviour (travelling, searching or eating) and habitat substrate (sand, patchy algae or rocky reef) were included as fixed effects and individual fish ID as a random effect. An interaction was included between the main effects of behaviour and substrate. Models were constructed using the *clmm* function in the ordinal package (Christensen, 2019). Likelihood‐ratio tests were used to test for the significance of the interaction and the fixed effects. The interaction term was dropped if it was not significant and the main effects were analysed. Significance of the main effects were obtained with an ANOVA type II deviance table using the *Anova.clmm* function in the RVAideMemoire package (Herve, 2020). The probabilities and 95% confidence intervals for goatfish adopting a particular colour morph when engaging in a particular behaviour, or on a certain substrate, were calculated using the *ggpredict* function from the ggeffects package (Lüdecke, 2018).

#### 2.5.2 Follower fish associations—following time

To assess whether the times spent by follower fish were influenced by the colour and/or behaviour of the focal goatfish, an approximation of total time that conspecifics and associated fish spent following focal fish were first obtained. The number of follower fish recorded at each time interval (every 5 s) was first aggregated and summed by individual focal goatfish, goatfish colour and behaviour to provide the total intervals that conspecifics or associated fish followed the focal goatfish. The total intervals were then multiplied by five (as each time interval was 5 s) to obtain an approximation of the total time (in seconds) that conspecifics and associated fish followed the focal goatfish. Linear mixed‐effects models were constructed in R using the lme4 package (Bates et al., 2015) with the follow time of conspecifics and associated fishes included as the response variables in two separate models. Focal goatfish colour (white, pale red or dark red) and behaviour (swimming, searching and eating) were included as fixed effects with individual focal fish ID as a random effect in both models. An interaction between colour and behaviour was also included in both models. The data were log (*x* + 1) transformed and the models met regression assumptions of linearity and homogeneity of variances with normal distribution of residuals. Log‐likelihood ratios were used to check for significance of the interaction. Pairwise comparisons for significant interactions were obtained using *glht* function in the multcomp package. The interaction term was dropped if it was not significant, and the main effects were analysed. Significance of the main effects were obtained with an ANOVA type III table using the *anova* function in the lmertest package (Kuznetsova et al., 2017). Pairwise comparisons among main effects were obtained using the pairwise method in the emmeans package (Lenth, 2022).

#### 2.5.3 Colour and behaviour of follower conspecifics

To evaluate whether the colour or behaviour of a conspecific was influenced by the focal goatfish colour, data were analysed using clmm models. As with focal fish, the colour exhibited by a conspecific was ordinal with three levels (white < pale red < dark red). We also included the behaviour of the conspecific as an ordinal factor (travelling < searching < eating). We constructed clmm models using the *clmm* function in the ordinal package in R. Response variables were conspecific colour or conspecific behaviour, the fixed effect was the colour of the focal fish in both models with the individual focal fish included as a random effect in both models. To tease out whether the behaviour of the focal fish was a driver in behaviour of follower fish, we constructed a clmm model with conspecific behaviour as the response variable and focal fish behaviour as the fixed effect. Individual fish were included as the random effect. In all models, likelihood‐ratio tests were used to test the significance of fixed effects. Probabilities and 95% confidence intervals for conspecifics adopting a particular colour or behaviour depending on the colour of the focal fish were calculated using the *ggpredict* function from the ggeffects package (Lüdecke, 2018).

#### 2.5.4 Behaviour of heterospecific follower fish

The behaviour of other heterospecific follower fish in response to focal goatfish colour and behaviour were analysed using clmm models using follower fish behaviour as an ordinal response term. In the models, the strongest response was eating with travelling as the weakest response (travelling < attracted < eating). Models were constructed using the same approach as outlined for the behaviour of conspecific followers above.

## 3 RESULTS

### 3.1 Rate of *U. lineatus* colour change

We followed 35 individual goatfish for 3 min each, within which we observed 38 occurrences of *U. lineatus* rapidly changing from the neutral white colour to the dark red banded pattern. We observed that *U. lineatus* changed colouration from neutral white to the pale red colouration in 1.92 ± 0.1 (mean ± 1 SE) s. Individuals changed from the white to the dark red banded patterning in 6.19 ± 0.3 s. At the completion of a foraging event, when the fish was no longer in the nose‐down position or actively chewing, it rapidly shifted back to the white colouration. We did not observe any occurrences where the fish transitioned from dark red to pale red following an active foraging event. The time for goatfish to change from the dark red pattern back to white was 2.52 ± 0.1 s.

### 3.2 Context of *U. lineatus* colour change

**FIGURE 3:**
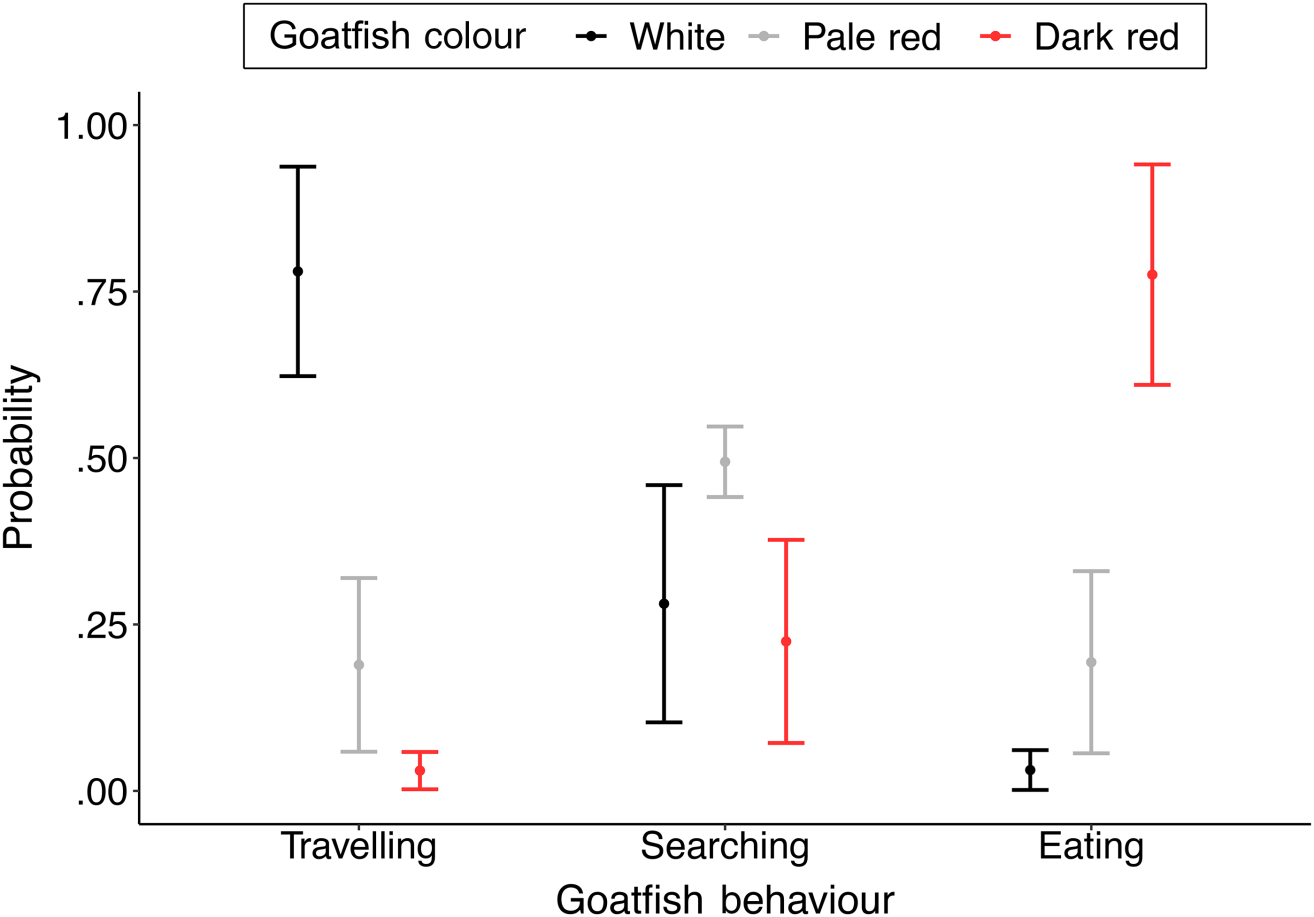
Probability of *Upeneichthys lineatus* adopting a particular colouration depending on its behaviour. Whiskers represent 95% confidence intervals (*n* = 35 individual tracks).

Of the total observation period (all 35 tracks combined), *U. lineatus* displayed the white colouration 37.3%, the dark red colouration 38.9% and the pale red 23.8% of the time. The goatfish were travelling 28.1%, searching 63.7% and eating for 8.1% of the observation time. No significant interaction was observed between the behaviour and the underlying substrate on the probability of *U. lineatus* displaying a particular colour (X ^2^ = 3.769, *df* = 4, *p* = .438). Analysis of the main effects showed no impact of substrate on *U. lineatus* colour (Χ ^2^ = 2.385, *df* = 2, *p* = .303). However, their behaviour did have a significant effect on how likely *U. lineatus* were to adopt a particular colour (Χ ^2^ = 310.641, *df* = 2, *p* < .0001). When travelling, the probability of *U. lineatus* adopting the white colouration was 78.5% (95% confidence intervals (CI) = 62.9, 94.1), significantly higher that the probability of adopting the pale red (18.6%, 95% CI = 5.6, 31.5) or dark red (3.0%, 95% CI = 0.2, 5.7) colour display. When *U. lineatus* was searching for food, the probability of displaying a pale red colouration was 49.5% (95% CI = 44.4, 54.7), significantly higher than the probability of adopting a white (27.6%, 95% CI = 9.9, 45.3) or dark red colouration (22.9%, 95% CI = 7.3, 38.4). When goatfish were actively eating, the probability of adopting dark red colouration was 77.3% (95% CI = 60.5, 94.0), significantly higher than adopting the pale red (19.6%, 95% CI = 5.7, 33.4) or white colouration (3.2% 95% CI = 0.1, 6.2; Figure 3).

### 3.3 Follower fish associations—following time

**FIGURE 4:**
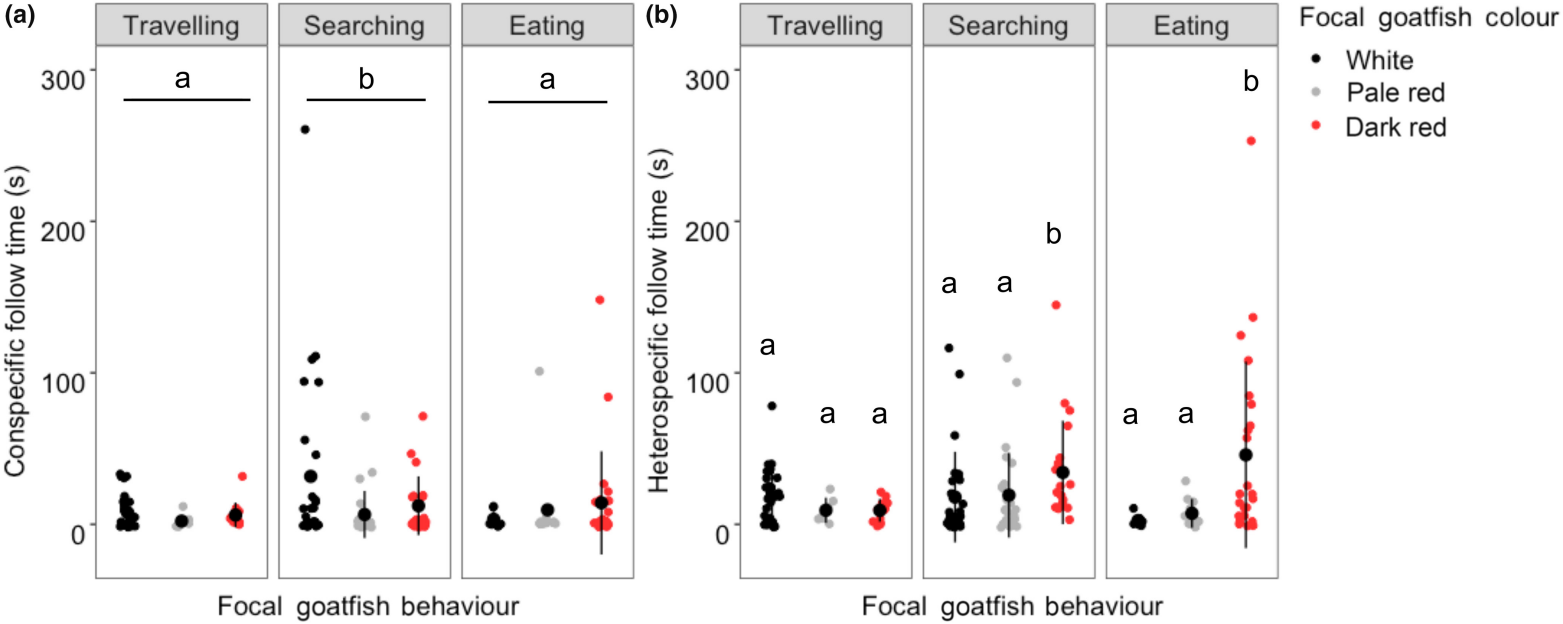
Mean (±SE) time that (a) conspecifics and (b) heterospecifics spent following focal *U. lineatus* while displaying the different behaviours and colour patterns. Data points demonstrate time spent interacting by individual fish. Differences in lower case letters indicate significant difference (*p* < .05) in following time (*n* = 35 individual tracks).

Conspecifics were observed frequently with the focal goatfish being in 32.1% of the individual tracks, which was second only in frequency to *O. lineolatus*. No significant interaction was observed between focal goatfish colour and behaviour on following time of the conspecifics (*X* ^2^ = 4.747, *df* = 4, *p* = .314). Analysis of the main effects showed the focal goatfish colour did not affect following time of conspecifics (*F* = 1.135, *p* = .343), but there was a significant effect of the focal goatfish behaviour on the conspecific's follow time (*F* = 3.773, *p* < .026). Conspecifics spent significantly more time following focal goatfish that were searching compared with those that were travelling (*t* = 2.449, *p* = .041). There was no difference in the amount of time conspecifics spent following focal fish when they were eating compared with searching (*t* = −1.997, *p* = .117) or travelling (*t* = 0.247, *p =* .967; Figure 4a).

In total, we observed 16 different species of fish associating with focal *U. lineatus*. The most observed follower fish was the southern Maori wrasse (*Ophthalmolepis lineolatus*), occurring in 32.4% of all individual tracks. The heterospecific fishes were generally resident fishes that typically inhabit inshore rocky reefs. Of the 17 species observed, 13 are carnivores with a diet predominately of benthic invertebrates, two are omnivores with a diet of algae and benthic invertebrates, and one species, the mado (*Atypicaths stigatus*), is predominately a planktivore with reports suggesting they are often attracted to benthic feeders due to the potential disturbance of a food source into the water column (Glasby & Kingsford, 1994; a complete list of follower fish and following times can be found in Appendix S2). A significant interaction between focal fish colour and behaviour on the following time of heterospecific follower fishes was observed (Χ ^2^ = 13.077, *df* = 4, *p =* .011). When focal fish were eating, heterospecifics spent significantly more time interacting with them when the focal fish was dark red compared with pale red (*z* = 2.864, *p* = .011) or white (*z* = 2.813, *p* = .013). There was no difference in interaction between heterospecifics and focal fish when the focal individual was between pale red and white (*z* = 0.495, *p* = .872). When focal fish were searching, heterospecifics spent significantly longer interacting when the focal fish were dark red compared to white (*z* = 3.198, *p* = .004) or pale red (*z* = 2.539, *p =* .030). However, no difference between the pale red and white colour (*z* = 0.744, *p* = .737) when searching was observed. There were no differences in the time heterospecifics spent following the focal fish when it was travelling regardless of its colour (dark red vs. white *z* = −1.045, *p* = .544; dark red and pale red *z* = −0.297, *p* = .952; white vs. pale red *z* = −0.297, *p* = .952; Figure 4b).

### 3.4 Colour and behaviour of conspecifics

Follower conspecifics were significantly more likely to adopt the white colouration regardless of the focal goatfish colour (Χ ^2^ = 6.335, *df* = 2, *p* = .042). Conspecifics were also most likely to engage in search behaviour around focal goatfish irrespective of focal fish colour (Χ ^2^ = 27.102, *df* = 2, *p* < .001). The likelihood of follower conspecifics engaging in a particular behaviour was influenced by the behaviour of the focal goatfish (Χ ^2^ = 131.85, *df* = 2, *p* = <.001). When focal goatfish were travelling, conspecifics were most likely to also adopt travelling behaviour rather than searching or eating. When focal goatfish were searching or eating, the probability of conspecifics also searching was significantly higher than travelling or eating (Appendix S3; Table 2).

**TABLE 2:** Probability, standard error (1 SE) and 95% confidence intervals (95% CI) for the follower conspecifics adopting a particular colour or behaviour depending on the focal goatfish colour or behaviour.

	Focal fish	Follower fish	Probability (%)	SE	95% CI	
Focal fish colour Follower conspecific colour	White	White	96.5	4.1	88.5	104.4
	White	Pale red	3.2	3.7	−4.1	10.5
	White	Dark red	0.3	0.4	−0.4	1.0
	Pale red	White	88.6	12.3	64.5	112.7
	Pale red	Pale red	10.4	11.1	−11.4	32.2
	Pale red	Dark red	1.0	1.2	−1.4	3.4
	Dark red	White	95.4	5.3	85.0	105.8
	Dark red	Pale red	4.2	4.9	−5.3	13.7
	Dark red	Dark red	0.4	0.5	−0.5	1.3
Focal fish colour Follower conspecific behaviour	White	Travelling	33.5	8.8	16.2	50.7
	White	Searching	64.2	8.0	48.6	79.9
	White	Eating	2.3	1.0	0.3	4.3
	Pale red	Travelling	10.3	4.5	1.5	19.2
	Pale red	Searching	80.3	2.5	75.5	85.2
	Pale red	Eating	9.3	4.2	1.0	17.6
	Dark red	Travelling	7.2	3.1	1.2	13.2
	Dark red	Searching	79.5	3.2	73.2	85.9
	Dark red	Eating	13.3	5.2	3.2	23.4
Focal fish behaviour Follower conspecific behaviour	Travelling	Travelling	77.2	7.7	62.1	92.3
	Travelling	Searching	22.7	7.6	7.7	37.7
	Travelling	Eating	0.2	0.1	0.0	0.3
	Searching	Travelling	6.9	2.4	2.1	11.6
	Searching	Searching	86.6	2.2	82.3	90.9
	Searching	Eating	6.5	2.3	2.0	11.0
	Eating	Travelling	2.6	1.3	0.1	5.1
	Eating	Searching	81.4	5.2	71.2	91.6
	Eating	Eating	16.0	6.1	4.1	27.9

### 3.5 Behaviour of heterospecific follower fishes

Both the colour and behaviour of the focal goatfish had a significant effect on the likelihood of heterospecific follower fishes displaying a particular behaviour (colour: Χ ^2^ = 99.639, *df* = 2, *p* < .001, behaviour: Χ ^2^ = 85.927, *df* = 2, *p* < .001). When the focal fish was white, heterospecifics were more likely to be travelling or showing attraction to the focal fish than eating. When focal goatfish were pale red, the probability of heterospecifics displaying attraction was significantly higher than the probability of heterospecifics travelling or eating. When focal goatfish were dark red, the probability that the heterospecific followers would show attraction to the focal fish was significantly higher than the probability of them travelling, they were also more likely to feed around dark red focal fish than travel.

When the focal fish was travelling, the probability of heterospecifics also travelling was significantly higher than them showing attraction or eating. In both cases of the focal fish searching or eating, the associated fish were significantly more likely to display attracted behaviour than travelling or eating. In both cases, there was no difference between attraction and eating when focal fish were searching or eating. All probabilities and confidence intervals are outlined in Table 3.

**TABLE 3:** Probability, standard error (SE) and 95% confidence intervals (95% CI) for the follower heterospecifics adopting a particular colour or behaviour depending on the focal goatfish colour or behaviour.

	Focal fish	Follower fish	Probability (%)	SE	95% CI	
Focal fish colouration Follower heterospecific behaviour	White	Travelling	57.7	6.0	46.0	69.3
	White	Attracted	39.6	5.3	29.2	50.1
	White	Eating	2.7	0.8	1.2	4.2
	Pale red	Travelling	18.0	4.0	10.1	26.0
	Pale red	Attracted	67.2	2.2	63.0	71.5
	Pale red	Eating	14.7	3.5	7.9	21.6
	Dark red	Travelling	9.3	2.1	5.3	13.4
	Dark red	Attracted	63.7	3.1	57.6	69.7
	Dark red	Eating	27.0	4.4	18.3	35.7
Focal fish behaviour Follower heterospecific behaviour	Travelling	Travelling	60.5	6.2	48.4	72.5
	Travelling	Attracted	36.9	5.5	26.1	47.7
	Travelling	Eating	2.6	0.7	1.1	4.1
	Searching	Travelling	17.7	3.2	11.5	23.9
	Searching	Attracted	66.4	2.1	62.3	70.5
	Searching	Eating	15.9	2.9	10.2	21.7
	Eating	Travelling	13.3	2.9	7.7	19.0
	Eating	Attracted	65.7	2.4	61.1	70.4
	Eating	Eating	21.0	4.0	13.0	28.9

## 4 DISCUSSION

*Upeneichthys lineatus* can display rapid and dynamic colour change, from white to a dark red banded pattern, with colour and pattern change associated with foraging behaviour. Moreover, this dynamic colour change appears intentionally linked to foraging behaviour for the focal fish, and can direct the behaviours of the following fishes. *U. lineatus* travelling or swimming between locations are most likely to exhibit a plain white colouration. When actively feeding with their heads in the sediment, however, individuals are more likely to adopt a distinct dark red banded pattern. A pale red banded colouration was mostly observed when *U. lineatus* were searching in sediment for food, and it is possibly a transient colour between travelling and actively feeding. Focal *U. lineatus* regularly had both conspecifics and other species following them. The dark red patterning of the focal goatfish did not appear to influence the follow time of conspecifics, rather they spent more time around focal goatfish that were searching, irrespective of colouration. Conspecifics were most likely to be white in colour and adopt searching behaviour regardless of the focal fish colour or behaviour. Other species of follower fish responded to changes in colour and pattern of the focal fish with follower fish spending significantly more time interacting with *U. lineatus* that were exhibiting the dark red patterning while either eating or searching for food. Other fish were more likely to show attraction to focal fish displaying the pale red and dark red patterning, although this is difficult to tease apart from the behaviour. The high likelihood of *U. lineatus* adopting the dark red patterning when feeding and the increased time that associated fish spend following them when red suggests colour change plays a role in interspecific signalling. These results indicate that rapid colour change in teleost fish might be used to communicate information relating to food resources within and between species, providing goatfish with increased protection via a safety‐in‐numbers approach.

### 4.1 Ecological drivers of colour change

It is possible that the head‐down feeding position of *U. lineatus* places it in a compromised position in terms of identifying danger and escaping predation. When feeding with obstructed vision the likelihood of danger increases, thus the red banded patterning may be a predator defence strategy. It is likely that the adoption of the red banded colouration is physiologically costly as any change to the arrangement or density of pigment organelles requires an expenditure of energy (Alfakih et al., 2022). These signalling costs may explain why *U. lineatus* adopts the red patterning only when there is perceived danger and reverts to a neutral white colour when travelling and not hindered from escape, a trade‐off between competing demands of ornamentation and fitness (Svensson & Wong, 2011; Zahavi, 1975). *U. lineatus* may be using colour change for camouflage, but the conspicuousness of the red banded pattern, which often bears no resemblance to the background (Phillips et al., 2017), deems background matching and disruptive camouflage unlikely. While red is commonly considered a warning signal in many environments (Blount & Mcgraw, 2008), in this scenario it is unlikely given the rapid absorption of red light in relation to ocean depth and that many temperate fish species lack visual pigments with sensitivity to longer wavelengths (Lythgoe et al., 1994). It is also unlikely that the contrasted light and dark banding is an aposematic signal as the only reports of aposematism in teleost fish are the spiny appendages found in puffer fish and lionfish (Caro & Ruxton, 2019). Alternatively, the dynamic colour change of *U. lineatus* may be a foraging or social cue to increase foraging success. While food acquisition signalling is often used by predators to increase hunting success of prey (Caro & Allen, 2017), it is feasible that *U. lineatus* use it to increase collective vigilance while in the compromised head‐down feeding position. Signalling to other fishes and the formation of mixed‐species groups can decrease the risk of predation, allowing an animal to decrease its level of vigilance (Périquet et al., 2010) and feed with greater efficiency (Morse, 1977).

### 4.2 Conspecific follower fish

Focal *U. lineatus* regularly had conspecifics following and interacting with them but colour change did not appear to be the driver of these interactions. Overall, conspecifics spent the most amount of time following focal goatfish when exhibiting search behaviour, regardless of colour. Conspecifics were more likely to adopt the white colouration and engage in search behaviour, again irrespective of the focal goatfish colour. The response of conspecifics to the search behaviours of focal fish is indicative of local enhancement, a process where the behaviour of an individual attracts another individual to a particular location (Brown & Laland, 2003), or in this case an advertisement of prospective food patches (Baird et al., 1991). Both shoaling fish (Reebs, 2000) and non‐shoaling fish (Brown & Laland, 2002) use local enhancement with behaviours such as darting movements or body positions to communicate information pertaining to food availability and it could be that conspecifics recognise the head‐down position as feeding. We did see more associated conspecifics engaged in search behaviour alongside focal fish when the focal goatfish were pale or dark red, but this was likely because *U. lineatus* are mostly likely to adopt the red colouration when searching and eating, and it is difficult to tease the drivers of this apart. Models assessing the influence of focal behaviour on the behaviour of follower conspeci (Appendix S3) show that follower fish are likely to undertake similar behaviour to the focal fish. When conspecifics interacted with focal fish, they were more likely to adopt a white colouration regardless of focal fish colour. It is possible that follower conspecifics select a different colour pattern when joining the focal goatfish that is already feeding in an abundant food patch. Alternatively, it could be a difference in developmental stages between fish with younger fish following more experienced goatfish. Given that *U. lineatus* likely have the capacity to perceive red colouration (Tosetto et al., 2021) it is possible that the red patterning adopted by the focal fish is a signal of dominance (Pryke, 2009) and there is social learning in *U. lineatus*. Further research is required to quantify whether colour also has a role in the social hierarchies of *U. lineatus*.

### 4.3 Heterospecific follower fish

Heterospecific followers interacted the most with *U. lineatus* displaying dark red patterning when they were both searching and eating, implying the dark red banding may be an interspecific signalling tool. Heterospecifics were more likely to show attracted behaviour towards the focal goatfish when they displayed the pale and dark red banding. However, as with the observations of follower conspecifics, this was comparable to when the focal goatfish were searching and eating, thus differentiating whether it is colour or behaviour of focal fish driving behaviour of follower fish is difficult. Nonetheless, heterospecific followers spent significantly longer interacting with focal *U. lineatus* when they were displaying the dark red patterning while searching and eating. It is important to note that many temperate marine species lack the visual pigments to detect longer wavelengths (Bowmaker, 1990), it is likely that some associated fishes might perceive this as a purely achromatic (black and white) signal, rather than a chromatic signal (red and white). Goatfish are regularly described as a nuclear species in multispecies foraging associations (Lukoschek & Mccormick, 2002). While group feeding may increase competition for resources, many studies suggest that follower fish will only target prey that are exposed or made available by goatfish suggesting competition for the same resources is minimal (Sazima et al., 2007). It is likely in this situation that multispecies group feeding may enhance the ability for some individuals to detect and feed on otherwise unobtainable prey without competing directly for the food preferred by *U. lineatus*. This enhanced prey detection occurs via behavioural cues of successful foragers (Lukoschek & Mccormick, 2002; Ryer & Olla, 1992). In the case of follower fishes, it is regularly reported that they are attracted to the sediment plumes created from the bottom disturbance of nuclear fish (Lukoschek & Mccormick, 2002; Sazima et al., 2007; Strand, 1988) more so than the visual appearance of the nuclear fish (Krajewski, 2009), but to date, assessment of foraging associations has only been undertaken in coral reefs, and there has been no consideration of colour and/or pattern change in any studies. In our study, we observed that *U. lineatus* produced little disturbance when searching for food suggesting that associated follower fishes were responding to the appearance of the fish rather than bottom disturbance in this system.

### 4.4 Rapid colour change as a foraging signal

Given that *U. lineatus* will readily adopt the dark red patterning when feeding, and associated fishes spend significantly longer following them when dark red, we suggest that their dynamic colour change plays a role in interspecific signalling. Locating and exploiting resources is a challenge for all organisms and given the patchy nature of benthic infauna (Kraan et al., 2009), the capacity to exploit resources is arguably even more important. Social foraging enhances the ability of some individuals in a group to locate and consume prey through the transfer of information about the location and or quality of food sources (Lukoschek & Mccormick, 2002; Ryer & Olla, 1992). A variety of signals are used by animals to indicate successful feeding. Honeybee (*Apis mellifera*) foragers perform a waggle dance to reveal the presence and location of profitable food sources to nest mates (Al Toufailia et al., 2013), cliff swallows (*Hirundo pyrrhonota*) use a vocal ‘squeak call’ to alert conspecifics of food discovery (Brown et al., 1991), and it was recently discovered that day octopus (*Octopus cyanea*) will signal associated fishes to commence cooperative hunting through posturing with changes to skin colour and texture (Bayley & Rose, 2020). To the best of our knowledge, the use of colour change by fishes themselves to signal the availability of food has not been documented. While co‐operation in animal social groups is often reported to be within species (Torney et al., 2011), there are multiple reports of multispecies feeding aggregations (Krajewski et al., 2006; Pryor & Milton, 2021; Sazima et al., 2006) and evidence that fish use referential gestures to form collaborations with other species of fish and invertebrates (Vail et al., 2013). Furthermore, we have seen that some species of goatfish use colour change in mutualistic cleaning interactions with cleaner shrimp (*Ancylomenes pedersoni*) on a Caribbean Reef (Caves, Green, & Johnsen, 2018), demonstrating that goatfish do use colour change for interspecific signalling.

For a signalling system to arise and persist, the signal must, on average, increase the fitness of both the sender and the receiver Caves, Brandley, and Johnsen (2018). The signal should also be reliable, that is, a property of the signal correlates consistently with a characteristic of the signaller, and on receiving the signal, there should be a resulting change in state that benefits the receiver. The dark red patterning of *U. lineatus* as a daytime foraging signal meets these criteria. The associated fish (receivers) of the signal potentially benefit by obtaining information about food patch locality and gain access to otherwise unobtainable food. The focal fish (sender) benefits from increased predator detection, allowing more time to forage in the substrate and increasing their capacity to track food resources (Torney et al., 2011). Furthermore, the dark red pattern consistently correlated with the focal fish feeding and there is a change in state of the receiver, which is to follow the goatfish and presumably to obtain additional food. While the findings from this study do not conclusively demonstrate that associated fish are feeding more due to the dark red pattern, the increased time around the dark red fish suggests at the very least a positive response to the signal.

Whether it is the red colouration or the vertical banding of *U. lineatus* that animals are responding to is not known. While red colouration is used in many animals for sexual signalling and competitive interactions, it is not extensively found in the marine environment. In fish inhabiting marine environments, signalling often occurs via high contrast, achromatic vertical banding (Berglund & Rosenqvist, 2001; Erisman & Allen, 2005; Miller & Allen, 2006). *Upeneicthtys lineatus* can perceive the red colouration of conspecifics where red light is available and can possibly perceive the patterning from up to seven metres away in clear waters (Tosetto et al., 2021). The visual systems of the associated fishes identified in this study have not been studied to date, but given that many temperate coastal fish possess pigments tuned to the blue‐green regions of the spectrum, it is possible that they are unable to perceive changes in the red colouration of the focal fish and instead rely on the change in luminance or the striped patterning as a signal; however, without comprehensive study into the visual systems of associated fishes this remains inconclusive. Nevertheless, future studies that incorporate models to determine whether associated fishes are responding to changes in colour and/or pattern should be undertaken. Furthermore, the colour change of *U. lineatus* attracts other benthic carnivores to create temporary foraging associations, which may shift predation intensity from one patch of resources to another. The spatial and temporal variability of these foraging associations may influence benthic community organisation and patch dynamics (Hines et al., 1990; McCormick, 1995). Links between dynamic colour change and broader community functionality should be investigated. The breadth of communication systems used by fish must be explored to understand how fish have evolved behaviours to suit environmental stressors. Fish communication is an exciting and burgeoning area in both animal behaviour and cognition. Research suggests that fish can collaborate and communicate in ways previously thought to be limited to higher order vertebrates, such as humans and primates (Vail et al., 2014). It is likely that teleost fish are using rapid colour change as a signalling tool and understanding in which capacity this tool is displayed is important in further understanding the significance and evolution of this behaviour.

## AUTHOR CONTRIBUTIONS

**Louise Tosetto:** Conceptualization (equal); data curation (lead); formal analysis (lead); funding acquisition (equal); investigation (lead); methodology (equal); project administration (equal); resources (equal); software (equal); visualization (equal); writing – original draft (lead); writing – review and editing (equal). **Nathan S. Hart:** Formal analysis (supporting); funding acquisition (equal); investigation (supporting); methodology (supporting); project administration (equal); resources (equal); supervision (equal); validation (equal); writing – review and editing (equal). **Jane E. Williamson:** Conceptualization (equal); formal analysis (supporting); funding acquisition (equal); investigation (supporting); methodology (equal); project administration (equal); resources (equal); supervision (equal); validation (equal); writing – review and editing (equal).

## FUNDING INFORMATION

This study was funded through the Holsworth Wildlife Research Endowment Fund and the Department of Biological Sciences (now School of Natural Sciences) and Macquarie University.

## CONFLICT OF INTEREST STATEMENT

The authors have no competing interests to declare.

## Supporting information


Appendix S1‐S2:

Appendix S3:

Table S1:

Video S1:

Video S2:

Video S3:

Video S4:

## Data Availability

Datasets will be publicly available when the article is published. Data have been uploaded to the FigShare repository and are under embargo until publication: https://figshare.com/s/02385a402358a874e0c0. The https://doi.org/10.25949/22697368 will become active when the item is published.
