# Supplementary material for: Dynamic colour change as a signalling tool in bluelined goatfish (Upeneicthtys lineatus)
Source: Ecol Evol. 2023 Aug 25;13(8):e10328. doi: 10.1002/ece3.10328 (PMC10450840; doi:10.1002/ece3.10328)
Supplement: Supplementary file 1 — Appendix S1‐S2: [file ECE3-13-e10328-s004.docx]

APPENDIX 1


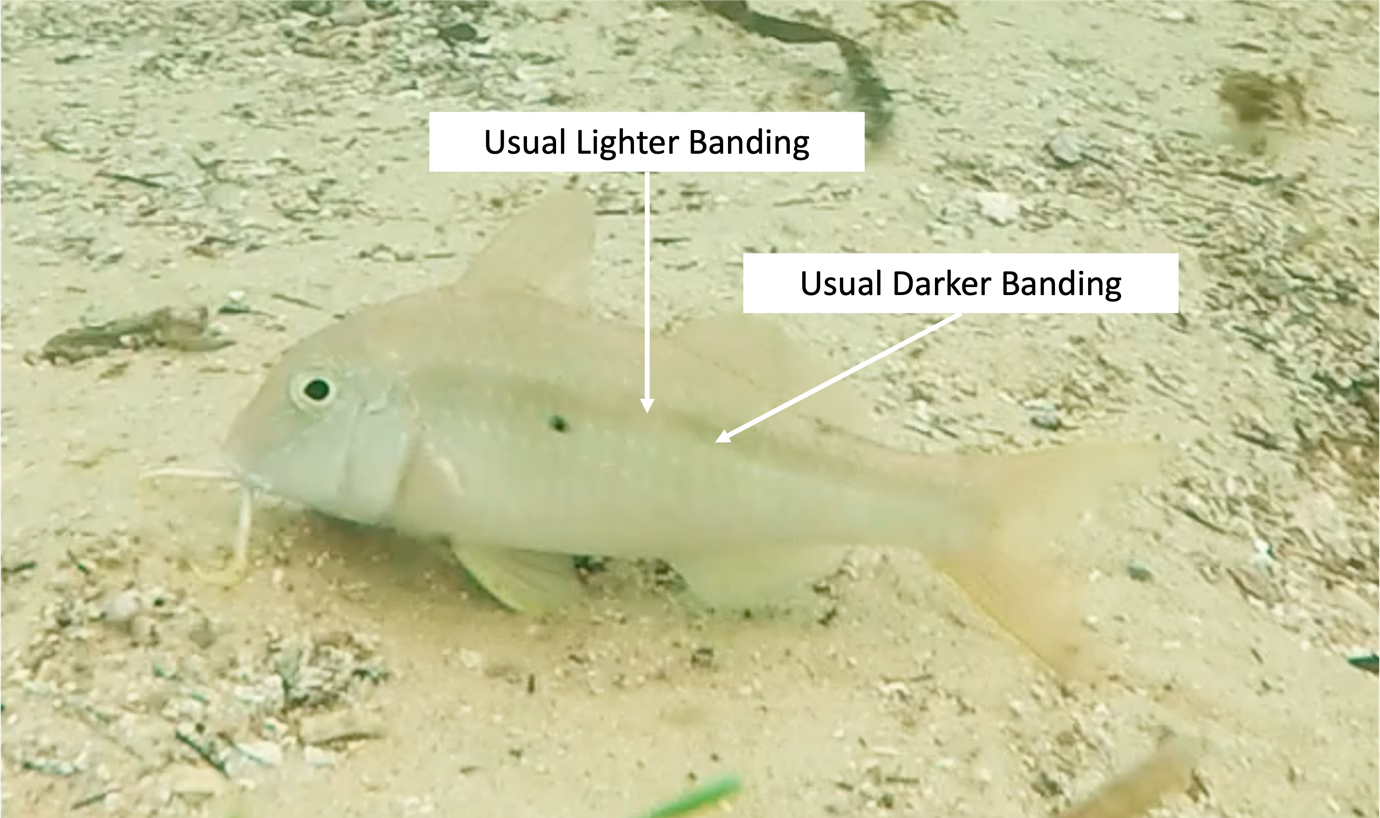


Calculating contrast values for goatfish displaying the white (neutral colouration). The arrows show the two locations measured where the bands would usually be apparent.

APPENDIX 2

Follower fish species and the total time in seconds that each species was found interacting with focal *U. lineatus*.

| ***Scientific Name*** | **Common Name** | **Forager** | **Diet** | **Habitat** | **Total Time (s)** |
| --- | --- | --- | --- | --- | --- |
| *Ophthalmolepsis lineolate* | Southerm Maori Wrasse | C | BI | IRR | 1985 |
| *Upeneicthys lineatus* | Bluelined Goatfish | C | BI | IRR | 1970 |
| *Parupeneus spilurus* | Blacksaddle Goatfish | C | BI | IRR | 725 |
| *Atypichthys strigatus* | Mado | P | PK | IRR | 635 |
| *Pseudocaranx georgianus* | Silver Trevally | C | BI | IRR* | 415 |
| *Scobinichthys granulatus* | Rough Leatherjacket | C | BI | IRR, SGB | 220 |
| *Acanthopagrus australis* | Yellowfin Bream | C | BI | IRR | 160 |
| *Eupetrichthys angustipes* | Snakeskin Wrasse | C | BI | SS | 105 |
| *Morwong fuscus* | Red Morwong | C | BI | IRR | 90 |
| *Parma microlepis* | White-Ear | OM | ALG, IN | IRR | 55 |
| *Enoplosus armatus* | Old Wife | C | BI | IRR | 55 |
| *Pictilabrus laticlavius* | Senator Wrasse | C | BI | IRR | 40 |
| *Gerres subfasciatus* | Silver Biddy | C | BI | SS | 20 |
| *Achoerodus viridis* | Blue Grouper | C | BI | IRR | 15 |
| *Upeneus tragula* | Bartail Goatfish | C | BI | SR | 10 |
| *Microcanthus strigatus* | Stripey | OM | ALG, IN | IRR | 5 |

Forager: C – Carnivore, P – Planktivore, OM – Omnivore. Diet: BI: Benthic Invertebrates, PK – Plankton, ALG - Algae, IN – Invertebrates. Habitat: IRR – Inshore Rocky Reef, SS – Sandy Substrate, SGB – Seagrass Bed, SR – Sandy Rubble (Tropical species).
