# Supplementary material for: Dynamic colour change as a signalling tool in bluelined goatfish (Upeneicthtys lineatus)
Source: Ecol Evol. 2023 Aug 25;13(8):e10328. doi: 10.1002/ece3.10328 (PMC10450840; doi:10.1002/ece3.10328)
Supplement: Supplementary file 2 — Appendix S3: [file ECE3-13-e10328-s007.pdf]

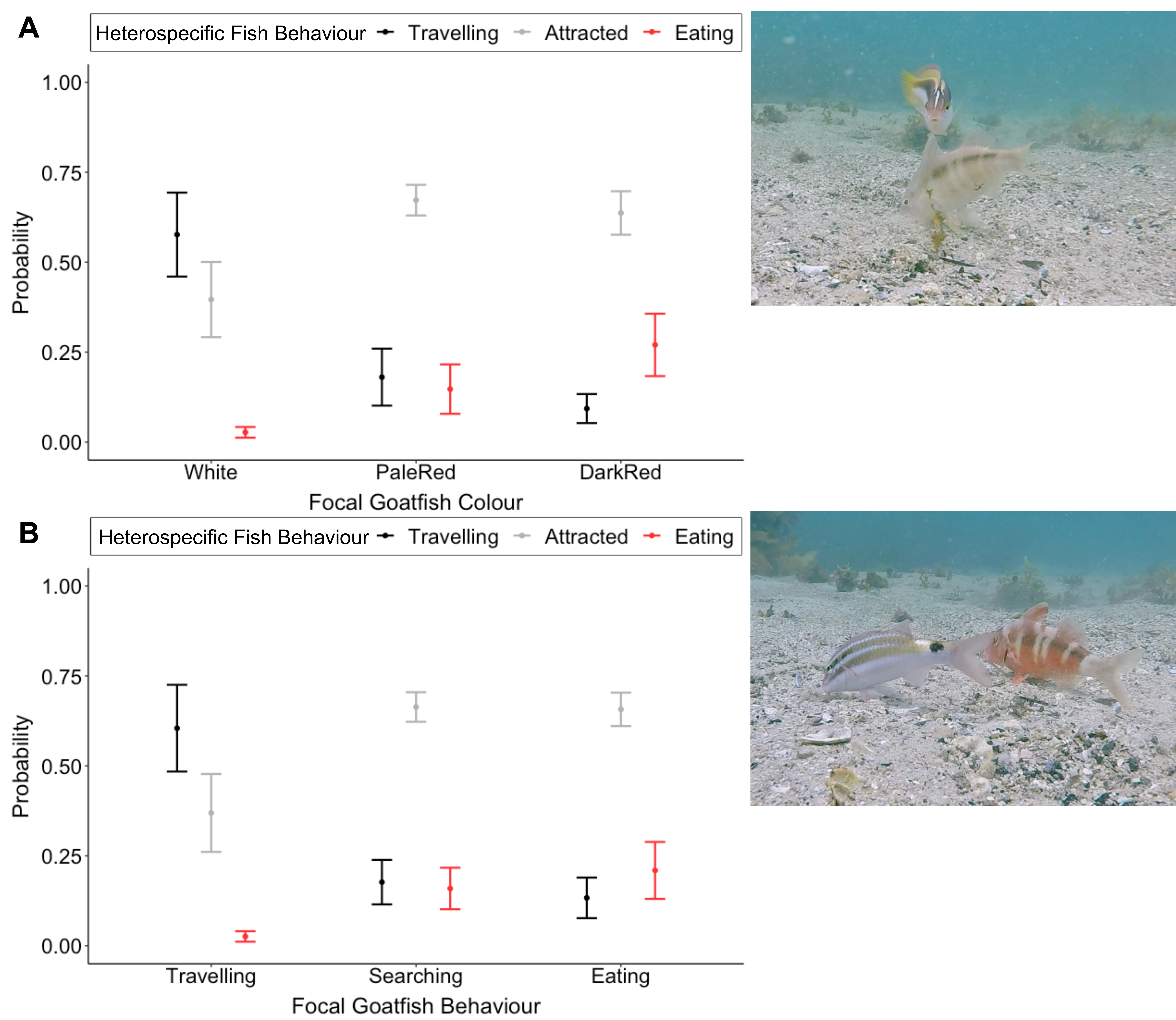
A
Heterospecific Fish Behaviour
Travelling
Attracted
Eating
Probability
1.00
0.75
0.50
0.25
0.00
White
PaleRed
DarkRed
Focal Goatfish Colour
B
Heterospecific Fish Behaviour
Travelling
Attracted
Eating
Probability
1.00
0.75
0.50
0.25
0.00
Travelling
Searching
Eating
Focal Goatfish Behaviour
