# Supplementary material for: Dynamic colour change as a signalling tool in bluelined goatfish (Upeneicthtys lineatus)
Source: Ecol Evol. 2023 Aug 25;13(8):e10328. doi: 10.1002/ece3.10328 (PMC10450840; doi:10.1002/ece3.10328)
Supplement: Supplementary file 3 — Table S1: [file ECE3-13-e10328-s006.docx]

**Supporting Information**

Dynamic colour change as a signalling tool in bluelined goatfish (*Upeneicthtys lineatus*)

Louise Tosetto, Nathan S. Hart & Jane E. Williamson

Corresponding author: Louise Tosetto – louise.tosetto@mq.edu.au

This file includes:

Table S1

Video S1 Description

Video S2 Description

Video S3 Description

Video S4 Description

Table S1. Follower fish species and the total time in seconds that each species was found interacting with focal U. lineatus.

| ***Scientific Name*** | **Common Name** | **Forager** | **Diet** | **Habitat** | **Total Time (s)** |
| --- | --- | --- | --- | --- | --- |
| *Ophthalmolepsis lineolate* | Southerm Maori Wrasse | C | BI | IRR | 1985 |
| *Upeneicthys lineatus* | Bluelined Goatfish | C | BI | IRR | 1970 |
| *Parupeneus spilurus* | Blacksaddle Goatfish | C | BI | IRR | 725 |
| *Atypichthys strigatus* | Mado | P | PK | IRR | 635 |
| *Pseudocaranx georgianus* | Silver Trevally | C | BI | IRR* | 415 |
| *Scobinichthys granulatus* | Rough Leatherjacket | C | BI | IRR, SGB | 220 |
| *Acanthopagrus australis* | Yellowfin Bream | C | BI | IRR | 160 |
| *Eupetrichthys angustipes* | Snakeskin Wrasse | C | BI | SS | 105 |
| *Morwong fuscus* | Red Morwong | C | BI | IRR | 90 |
| *Parma microlepis* | White-Ear | OM | ALG, IN | IRR | 55 |
| *Enoplosus armatus* | Old Wife | C | BI | IRR | 55 |
| *Pictilabrus laticlavius* | Senator Wrasse | C | BI | IRR | 40 |
| *Gerres subfasciatus* | Silver Biddy | C | BI | SS | 20 |
| *Achoerodus viridis* | Blue Grouper | C | BI | IRR | 15 |
| *Upeneus tragula* | Bartail Goatfish | C | BI | SR | 10 |
| *Microcanthus strigatus* | Stripey | OM | ALG, IN | IRR | 5 |

Forager: C – Carnivore, P – Planktivore, OM – Omnivore. Diet: BI: Benthic Invertebrates, PK – Plankton, ALG - Algae, IN – Invertebrates. Habitat: IRR – Inshore Rocky Reef, SS – Sandy Substrate, SGB – Seagrass Bed, SR – Sandy Rubble (Tropical species)

Video S1. A film clip of an individual *U. lineatus*, which provides an example of travelling behaviour.

Video S2. A film clip of an individual *U. lineatus*, which provides an example of searching behaviour.

Video S3. A film clip of an individual *U. lineatus*, which provides an example of eating behaviour.

Video S4. A film clip of an individual *U. lineatus* demonstrating its dynamic colour change. The goatfish shifts from a pale white which from a pale / buff white colouration to a more prominent vertically striped dark red pattern in seconds.

Copyright for the videos is held by Louise Tosetto
